# Waste Management: Valorisation Is the Way

**DOI:** 10.3390/foods10102373

**Published:** 2021-10-06

**Authors:** German Gemar, Ismael P. Soler, Eva M. Sánchez-Teba

**Affiliations:** 1 Department of Economics and Business Administration, University of Malaga, 29071 Malaga, Spain; emsanchezteba@uma.es; 2 Department of Applied Economics (Statistics and Econometrics), University of Malaga, 29071 Malaga, Spain; ipsoler@uma.es

**Keywords:** SciMAT, incineration, valorisation, waste-to-energy, municipal solid waste, bibliometric analysis

## Abstract

Waste management is one of the great problems in the world today. This study aims to analyse how scientific research has evolved in recent years in the field of waste management and what will be the key issues in the coming years, mainly in terms of recovery. The methodology used was longitudinal bibliometric analysis through scientific mapping using strategic maps and thematic networks. Among the findings, it was confirmed that the concept of incineration is fading due to social opposition and is changing to a much broader concept that encompasses it, such as valorisation. Being able to create a circular economy without waste should be the goal of policy makers. To achieve this, the waste hierarchy must be respected, which indicates that waste must be managed in this order: prevention, minimisation, reuse, valorisation, recovery and elimination.

## 1. Introduction

International development has brought serious threats, such as global warming and a shortage of fossil fuels due to the growing demand for fuels and overpopulation, thus requiring innovative and sustainable solutions [1,2,3]. The limitation of resources is already a fact; therefore, industries must increase the efficiency of their processes by improving them or looking for new uses for waste [1].

**Figure 1:**
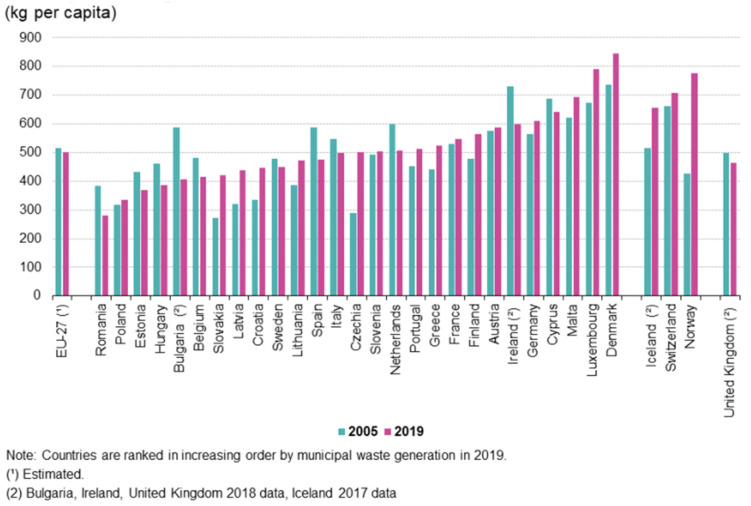
Municipal waste generated, 2005 and 2019. Source: Eurostat [7].

In accordance with the waste hierarchy defined in the Waste Framework Directive [4], waste management has evolved to a stronger focus on waste prevention, material recuperation, and recycling (paper, glass, metals, and others) [5]. Despite increasing attention on prevention and sustainability, total municipal solid waste (MSW) generation in the EU-27 increased from 198 million tons in 1995 to more than 225 million tons in 2019. A total of 502 kg of MSW per capita was generated in the EU in 2019, as shown in Figure 1.

On the other hand, United States, China, and India are the top three producers of municipal solid waste [6].

The composition of solid waste varies according to per capita gross domestic product. Organic waste is typical of low- or middle-income populations, whereas high-income populations produce more glass, paper, and metal waste [6]. This is why everything that is agreed to and legislated on waste in these countries and in the EU-27 will have a great impact worldwide.

**Figure 2:**
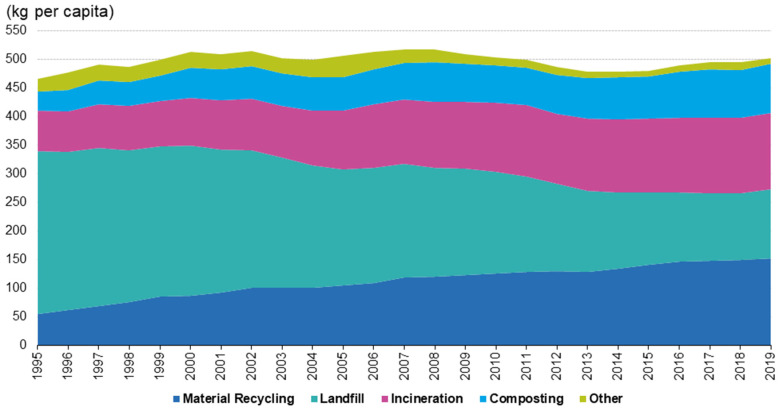
Municipal waste treatment, EU-27, 1995–2019. Source: Eurostat [7].

Figure 2 shows how municipal waste treatment in EU-27 has varied in the period 1995–2019. Unfortunately, more waste was generated per capita in this period than in 1995, and although the fraction sent to landfill is lower, it is still too much. In many parts of the world, landfilling is chosen for solid waste disposal. The organic waste in landfills undergoes physical, chemical, and biological transformations. This activity must be carried out in a sustainable way, so the optimal management of facilities, from their construction to their subsequent use, is critical, with special emphasis on the lining of the sanitary landfill, the thickness of the cover, the collection of leachate, and the recovery of gas [6].

Managing solid urban waste is a key activity, which includes the segregation of waste to enable recycling, conversion of waste into energy, incineration and composting or landfilling [6]. Waste from the food supply chain has great potential to be used in the production of fuel and some chemicals [1].

A total of 48% of MSW was recycled (material recycling and composting) [7], but the remaining quantity was made up of waste not easily separated or recovered (non-recyclable fraction) and required complex and expensive processes. This waste must therefore be transported to a controlled landfill site, where it does not cause harmful effects on the environment. Therefore, reducing the volume of waste, the recovery of waste and environmental protection will be subjects of primary concern for public authorities [6]. Some studies have focused on determining how to improve the environmental performance of certain materials. On study that stands out suggests using cane fibre as a substitute for fiberglass to reinforce transport pallets [8].

Existing practices regarding the reuse of municipal solid waste fly ash are also of concern to scientific research on waste. At this point, concern for construction materials (cement, ceramic, concrete, and glass), geotechnical applications (embankments and road pavement), agriculture, and sludge treatment is especially relevant. Each technology has its strengths and weaknesses, but the main objective must be to minimise waste and conserve resources [9].

Strategies for the recovery and reuse of food waste will be key in the coming years instead of conventional processing, i.e., incineration to recover energy, landfill, composting and the use of landfill gas.

To prevent waste from being taken to landfill, there are management systems to obtain benefits from it instead. This process is called the valorisation of residues.

In the contemporary scenario of (a) growing demand for fuels due to overpopulation and social changes, (b) global warming, (c) scarcity of fossil resources, and (d) need to minimise the use of landfills, it is necessary to develop sustainable and innovative strategies for the use of waste. Supply chain waste is a potentially significant resource to be used as raw material and/or energy. However, when it comes to using some techniques such as incineration, there is some public opposition.

The main objective of this study was, given the public opposition that the concept of incineration has had, to demonstrate that this concept has progressively been transformed into a friendlier one, such as the valorisation as energy, because it is a type of waste-to-energy process. For this, a bibliometric analysis was used, which confirmed this finding as the main conclusion.

## 2. Theoretical Background

Waste-to-energy incineration is an effective form of waste treatment and widely applied in many countries [10]. For example, Japan incinerates more than 80% of its MSW [10,11]. Incineration may use unsorted municipal waste or specific waste fractions. However, the main limitation to the use of incinerators is public opposition [10]. The public opposes the construction of these facilities [12] due to the production of emissions of particles, heavy metals, dioxins, etc., that are harmful to public health [13,14].

Problems related to health and environmental risks have been associated with incineration plants. However, good waste management must study what can and cannot be incinerated. For example, conventionally, food waste (FW) is dumped in a landfill site or incinerated. The elimination of FW is carried out by fermentation, composting, and pouring [15]. However, if incineration is chosen, the high moisture content of FW generates dioxins, and the landfill causes environmental and health problems, because it contributes to the generation of greenhouse gases [16]. To overcome these concerns, the recovery of materials for the production of products is an ideal solution, because FW can be converted into products such as biodiesel, methane, bioplastics, and organic fertilisers, among others [3].

Valorisation is a practice that adds economic value to waste while reducing the volume of waste that ends up in landfills.

Within the recovery of waste, the following are noted:

- Valorisation as energy (waste-to-energy), which is carried out using technologies that generate energy that comes from the materials containing this type of waste, although they also generate a small amount of waste that is difficult to eliminate. Technology will be the key, and thermochemical technologies such as incineration, gasification, pyrolysis, plasma technologies, or combinations of these will therefore be suitable for certain waste fractions [5,17];
- Valorisation as materials (waste-to-product), in which new materials are obtained, or a large portion of them are recycled, to avoid the use of new raw materials for the manufacture of materials. The materials that can be recovered are paper, glass and organic material. Composting serves to valorise organic material [5].

## 3. Materials and Methods

### 3.1. Data Collection

**Figure 3:**
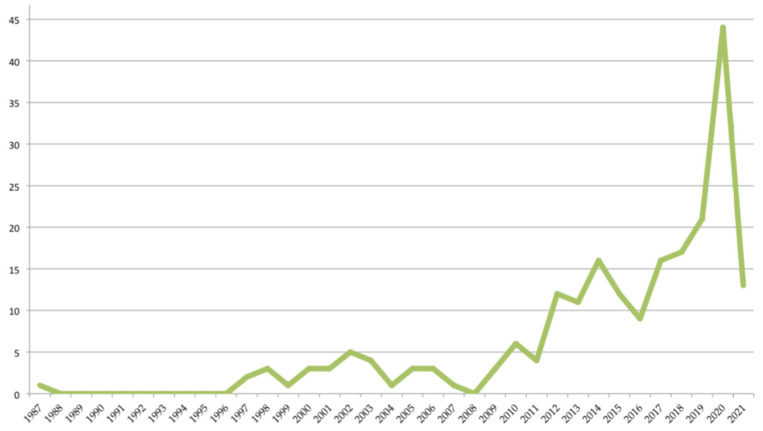
Number of documents per year of publication.

The Core Collection of the Web of Science [18] database was used in the present study, which searches leading academic journals and proceedings from around the world. It includes the Science Citation Index Expanded (SCIE), Social Sciences Citation Index (SSCI), Arts & Humanities Citation Index (AHCI), Emerging Sources Citation Index (ESCI), Conference Proceedings Citation Index (CPCI), Book Citation Index (BKCI) and Current Chemical Reactions and Index Chemicus. The objective was to analyse results to identify trends and publication patterns. It was interesting to know how the concept of incineration has evolved towards that of valorisation. In this work, the search was carried out on 11 March 2021, for the investigation period, which ran from January 1987 to 11 March 2021 and thus included all documents related to the study of ‘incineration’ and ‘valorisation’ treated simultaneously. To do this, we searched for ‘incineration’ AND ‘valorisation’ in the title, abstract, author keywords, and keywords plus. A total of 214 documents were obtained from 889 authors. Figure 3 shows the number of documents obtained in each year.

### 3.2. Methodology

The open-source software SciMAT (Science Mapping Analysis Software Tool) was used to perform a longitudinal analysis of scientific mapping. In this study, there were three periods: period 1, 1987–2010, 39 documents; period 2, 2011–2015, 55 documents; period 3, 2016–2021, 120 documents. Table 1 reports the number of documents per period.

To carry out the analysis, 10 steps have been followed:

1. Periods: 1987–2010; 2011–2015; 2016–2021;
2. Unit of analysis: words (author’s words, source’s words and added words);
3. Data reduction: not necessary in this analysis;
4. Kind of matrix: co-occurrence;
5. Network reduction: not necessary in this analysis;
6. Normalisation: equivalence index;
7. Clustering algorithm: simple centres algorithm; maximum network size: 12, and minimum network size: 3;
8. Document mapper: core mapper;
9. Quality measures: h-index, average citations, sum citations;
10. Longitudinal: evolution map: inclusion index; overlapping map: Jaccard’s index.

**Figure 4:**
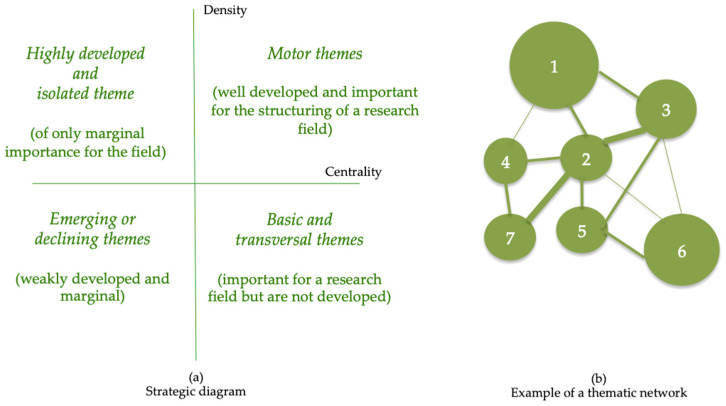
Strategic diagram and thematic network. Source: adapted from Cobo [19]. (**a**) Strategic diagram; (**b**) Example of thematic network.

A co-word analysis was carried out. In a co-word analysis, clusters are obtained that are considered ‘themes’. Each research topic is characterised following two parameters, namely, density and centrality, obtaining a classification of four groups, one in each quadrant, as follows (Figure 4a):

- ‘Motor themes’ are located in the upper right quadrant. They are well developed and important topics for structuring a field of research;
- Basic and transversal themes are located in the lower-right quadrant. Cross-cutting and basic themes are located there;
- Highly developed and isolated themes are located in the upper-left quadrant. They are subjects of marginal importance for the field, because they present external links without importance, although they maintain well-developed internal links;
- Emerging or declining themes are in the lower left quadrant. They are marginal, underdeveloped, emerging, or missing issues.

Thematic networks are formed from the keywords and their interconnections by drawing a network graph. The network is tagged with the name of the most central keyword of the topic (Figure 4b). The volume of the spheres is proportional to the number of documents of each keyword, and the width of each link between two spheres is proportional to the equivalence index.

**Figure 5:**
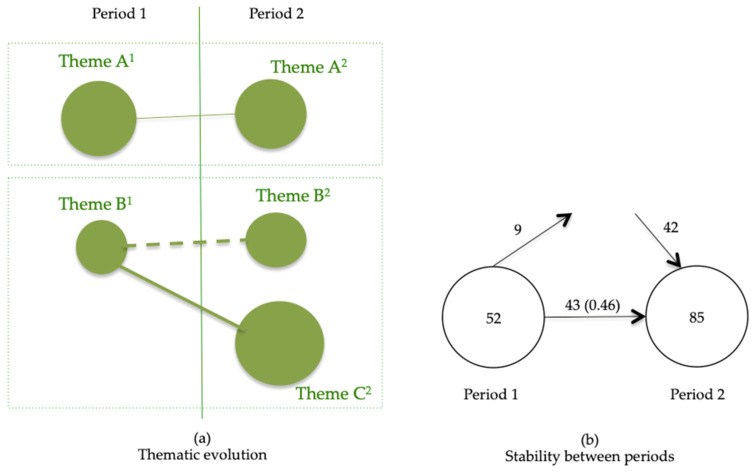
Examples of thematic evolution. Source: Source: adapted from Cobo [19]. (**a**)Thematic evolution; (**b**) Stability between periods.

In Figure 5a, two thematic areas framed in a rectangle are presented: one thematic area composed of Theme A^1^ and Theme A^2^, and another thematic area composed of Theme B^1^, Theme B^2^ and Theme C^2^. The thematic evolution map (Figure 5a) is the longitudinal representation of the work, and it is composed of as many columns as periods which have been chosen in the analysis: in our case, three. Each column contains the most relevant topics of that period (in the example there are two thematic lines). The first thematic line (theme A, that does not change its name from period 1 to 2). The second thematic line, theme B, begins in period one (theme B^1^), but nevertheless, in period 2, it becomes two themes (a theme that preserves the old name, B, and a new thematic line, C, opens). The lines that connect with the themes correspond to the evolution of these themes over time.

In Figure 5b, the stability between two consecutive periods is shown. Each circle represents the number of keywords for that period. The horizontal arrow shows the number of keywords shared in the two periods with the index of similitude between parentheses. The entry arrow represents the number of new keywords, and the exit arrow represents the number of keywords that are no longer present in the current period but were present in the previous period.

## 4. Results and Discussion

Table 2 shows the journals with more than three documents included in the present study. The most used journal was *Waste Management*, in which 22 of the documents used were published.

**Figure 6:**
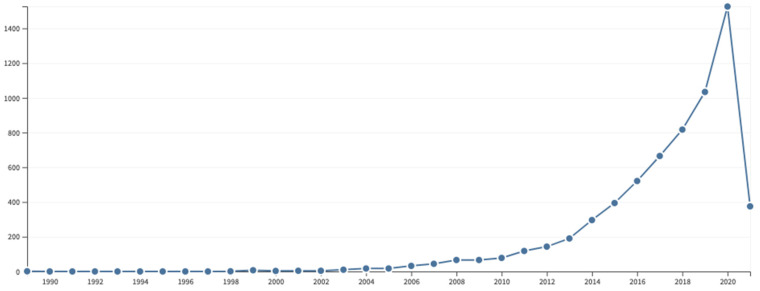
Sum of number of times cited per year.

Figure 6 shows the number of times per year that the articles included in the present study were cited. The subject aroused growing interest, recently reaching maximum interest. These articles had an h-index of 41; there were 41 articles with at least 41 citations. The total number of times cited was 6429, and without self-citation, 6286. They were cited in 5743 articles (5658 without self-citation).

Table 3 shows the number of citations of the main articles as well as the year of publication and the source where they were published. The most cited paper was “Food waste as a valuable resource for the production of chemicals, materials and fuels. Current situation and global perspective” [1], published in the journal *Energy & Environmental Science*.

### 4.1. Longitudinal Map

**Figure 7:**
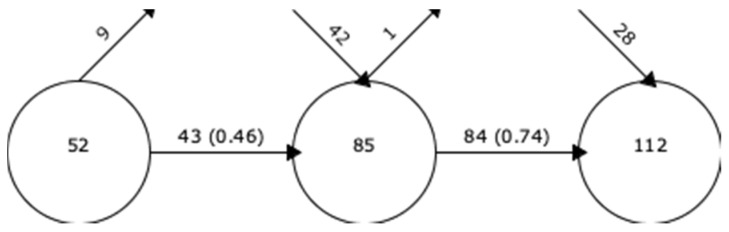
Stability between periods. Overlapping graph of keywords from 1987 to 2021.

To analyse the development of the present field of research, incineration and valorisation, it was useful to represent how these have evolved over time in different periods. Figure 7 shows a graph with circles that represent the periods described and the number of keywords of interest in those years. In the first period, from 1987 to 2010, there were 52 keywords. In the second period, there were 85 words, of which 43 words were from the previous period (9 words were no longer used), and 42 new words appeared. In the third period, there were 112 words, of which all but one came from the previous period, and 28 new words were incorporated. The stability index between the first two periods was 0.46. This stability index increased between the second and third period to 0.74. The increase in the stability index shows that the concepts treated were gradually consolidating, although they were still developing.

**Figure 8:**
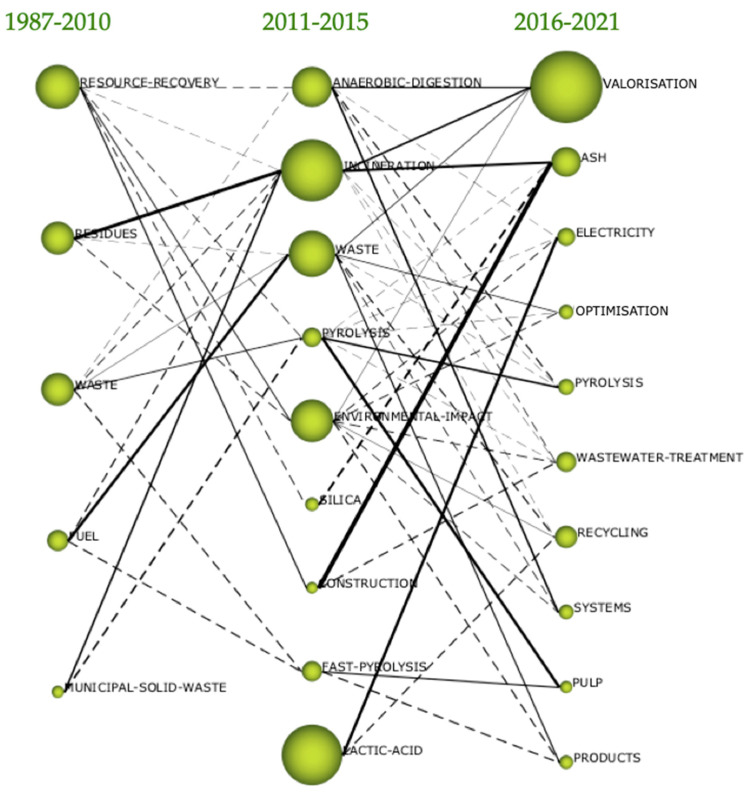
Thematic evolution map.

Regarding the evolution of the topics by periods, as seen in Figure 8, it was found that the concept of ‘valorisation’ was consolidated over time. In the period 1987–2010, the interest was in the recovery of resources. However, the ‘residues’ concept was gaining more prominence compared to the ‘waste’ concept.

In the period 2011–2015, the concept of incineration appeared strongly as a transformation from the previous period of the more generic concepts ‘waste’ and ‘municipal solid waste’. The concern in these years was that the recovery of waste from the previous period had not been enough, and that too much waste was still going to landfill, which is why the fundamental concern was in the concept ‘anaerobic digestion’ [25] as a transformation of the concepts ‘resource recovery’ and ‘waste’. Incineration represented a priority in these years. The generic concept ‘waste’ from the previous period was transformed into the concept ‘incineration’. In this second period, the waste treatment methods began to be concerned with their benefits and their costs. That is why studies on ‘pyrolysis’ and ‘fast pyrolysis’ were increasing. It was in this second period where the concept of ‘environmental impact’ took hold. In the third period, the main concern was valorisation.

The concept of ‘valorisation’ converged from the previous period of the concepts ‘anaerobic digestion’, ‘incineration’, ‘waste’ and ‘environmental impact’. In this third period, a concept also of interest was ‘ash’. On the one hand, the ‘incineration’ of the second period was called valorisation and the concern was ash. This concern for ash from incineration was especially relevant in ‘construction’, which is why the concepts of the Second Period of incineration and construction converged to the concept of ash as a current concern in scientific studies.

In the period 2016–2021, the main concept was valorisation, which linked the concepts of the previous period: incineration, waste and anaerobic digestion [17,33,34]. This is why it follows that the concept of valorisation is a more generic and friendlier concept; it did not produce rejection as the concept of incineration did. Researchers preferred the concept of ‘valorisation’, and yet in this period, 2016–2021, they attempted to avoid the concept of ‘incineration’ because it had a significant negative connotation with the general population, perhaps because this concept is associated with that of health risks.

### 4.2. Strategic Map and Thematic Network

Longitudinal analysis made it possible to know the evolution of the concepts between periods. In this section, the importance of each subject in the research field for each of the periods is analysed.

Table 4 shows the cluster properties, namely, the clusters for each period, indicating the score in terms of centrality, centrality range, density and density range. Likewise, Table 4 also shows the number of documents that used specific keywords as well as the h-index, average citations and the sum of citations.

#### 4.2.1. Period 1987–2010

**Figure 9:**
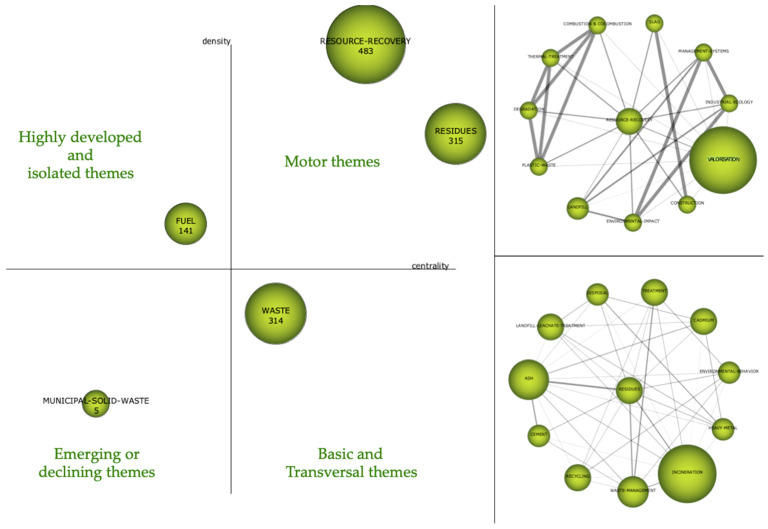
Strategic diagram and main thematic network, 1987–2010 period.

In Figure 9, a strategy map is shown on the left, and the main topics of that period are shown on the right. Regarding the strategic map, the most interesting topic is in the upper right quadrant, which is that corresponding to the ‘driving themes’. In this case, said driving themes are ‘Resource Recovery’ and ‘Residues’, with a centrality and density of 141.60–53.59 and 123.03–130.90, respectively. These two topics aroused the most interest of researchers in that period. In the lower right quadrant appears ‘Waste’, which is an important but basic topic. In the upper left quadrant, ‘Fuel’ appears as a highly developed theme with internal links but with unimportant external links. It can be said that it is a very specialised subject with a peripheral character. An emerging theme appears in the lower left quadrant, namely, ‘municipal solid waste’.

On the right, the network structure shows the relationship between the topics that make up the cluster, which is why two thematic networks corresponding to the main driving themes of that period are shown.

In the thematic network analysis of ‘Resource Recovery’, the following related keywords can be found: ‘valorisation’, ‘construction’, ‘environmental impact’, ‘landfill’, ‘plastic-waste’, ‘degradation’, ‘thermal treatment’, ‘combustion and co-combustion’, ‘resource recovery’ and ‘management systems’. However, ‘Valorisation’ is the most important theme, although it still does not become a central theme by itself but rather is associated with the concept of ‘resource recovery’.

#### 4.2.2. Period 2011–2015

**Figure 10:**
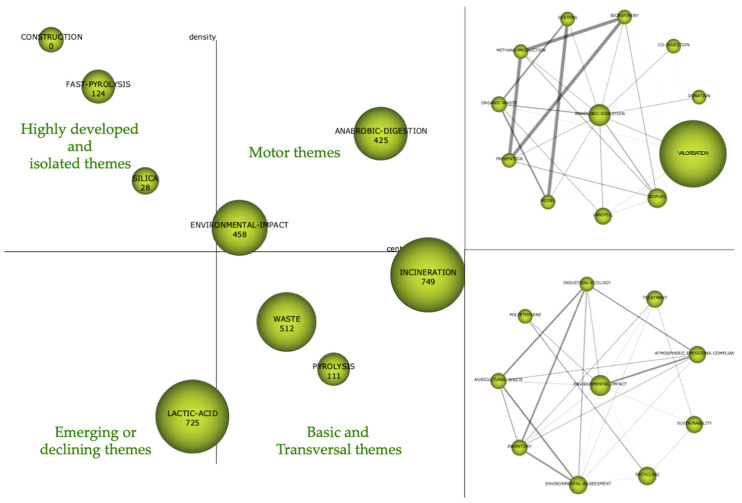
Strategic diagram and main thematic network, 2011–2015 period.

In Figure 10, the strategy map is shown on the left side, and the main topics of that period are shown on the right side. Regarding the strategic map, the most interesting topic is in the upper right quadrant, which is that corresponding to the ‘driving themes’. In this case, these themes are ‘Anaerobic digestion’ and ‘Environmental impact’ with a centrality and density of 156.89–66.29 and 94.29–52.01, respectively. These two topics aroused the most interest of researchers during that period. In the lower right quadrant, as in the previous period, ‘Waste’ appears again, which is an important but basic issue. It is important to point out that in this period, incineration appears as a basic and transversal issue that is starting to become a driving issue but does not quite achieve it. In the upper left quadrant, ‘Fast pyrolysis’, ‘Silica’ and ‘Construction’ appear as highly developed themes with internal links but unimportant external links. It can be said that they are very specialised and peripheral in nature. In the lower left quadrant, there is an emerging theme, namely, ‘Lactic acid’. The ecological conscience of society is increasing, which is why much of the research is dedicated to finding inexpensive organic carbon compounds for microbes to turn into valuable products. Agricultural and food waste is collected in large quantities all over the world to be incinerated, anaerobically digested and composted for the production of fertiliser, energy and heat. With the help of the bio-economy and the recovery of waste, biochemical products and materials can be formed, such as plasticiser based on lactic acid, fatty acids and succinic acid [35].

On the right side of Figure 10, the network structure shows the relationship between the topics that make up the cluster, which is why two thematic networks are shown corresponding to the main driving themes of that period.

In the thematic network analysis of ‘Anaerobic digestion’, the following related keywords can be found: ‘valorisation’, ‘biofuel’, ‘landfill’ and ‘co-digestion’, among others. However, ‘valorisation’ is the most important theme, although it has not yet become a central theme by itself but rather is associated with the concept of ‘Anaerobic digestion’. ‘Environmental impact’ is the second major theme around which there is a network with keywords such as ‘sustainability’, ‘recycling’, ‘environmental assessment’, ‘agricultural waste’, ‘treatment’ and ‘industrial ecology’, among others.

#### 4.2.3. Period 2016–2021

**Figure 11:**
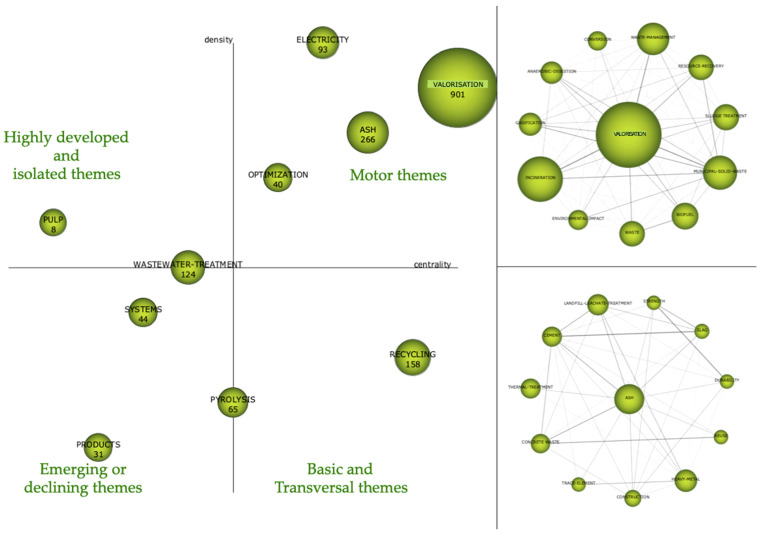
Strategic diagram and main thematic network, 2016–2021 period.

In Figure 11, the strategy map is shown on the left side, and the main topics of that period are shown on the right side. Regarding the strategic map, the most interesting topic is in the upper right quadrant, which is that corresponding to the ‘driving themes’. In this case, said driving themes are ‘valorisation’ [17,33,36], ‘ash’ [37], ‘electricity’ [3] and ‘optimisation’ [3] with a centrality and density of 197.17–39.92, 123.86–30.33, 102.02–58.44 and 90.14–29.55, respectively. These four topics aroused the most interest in researchers. In the lower right quadrant appears ‘recycling’, which is an important but basic issue. In the upper left quadrant, ‘pulp’ appears as a highly developed topic with internal links but with unimportant external links. It is a highly developed theme when it comes to food waste. It can be said that it is a very specialised subject with a peripheral character. Between the two left quadrants, ‘wastewater treatment’ appears as an emerging and widely developed topic. It is still an important issue that is expected to become a driving issue in the near future, given the interest of society in general in discovering new methods for wastewater treatment that entail less energy cost and that achieve final discharges without negative impacts on the environment. In the lower left quadrant, there are several emerging themes such as ‘products’ and ‘systems’, which are declining themes, and the ‘pyrolysis’ theme, which is between emergent and transversal themes.

On the right side of Figure 11, the network structure shows the relationship between the topics that make up the cluster, which is why two thematic networks are shown corresponding to the main driving themes of that period.

In the thematic network analysis of ‘valorisation’, the following related keywords can be found, among others: ‘incineration’, ‘waste management’ and ‘municipal solid waste’. However, ‘incineration’ is now within the network that leads the concept of ‘valorisation’, which is the subject most studied by researchers.

Ash is the second major theme around which there is a network with keywords such as ‘landfill’, ‘cement’, ‘thermal treatment’, ‘construction’, ‘heavy metal’ and ‘durability’, among others.

From the results, it is evident that the researchers are oriented towards the field of ‘valorisation’. This work has managed to broaden the knowledge by organising the literature from a longitudinal perspective, relating the concepts of ‘incineration’ and ‘valorisation’.

In addition to the keywords ‘valorisation’ and ‘incineration’, the most used keyword is ‘municipal solid waste’ (MSW), which is widely used when discussing incineration and valorisation. Research focuses on the municipal level, because it is the municipal administration that has the competence to manage the collection, transport, and treatment of MSW.

The next most used word is ‘waste management’. Optimal waste management is key for researchers. It is a concept that includes the collection, transport, treatment and disposal of waste, focusing on the waste management process and technologies, waste-related laws and economic mechanisms. Good waste management will also try to reduce the adverse effects of waste on human health, the environment and resources. By integrating the MSW concept with applied technologies and their measurement, the concept of the circular economy is reached.

Other widely used keywords include ‘ash’, which corresponds to a concern when mainly incineration is chosen.

‘Sludge treatment’ is also a research topic widely used in research and refers to sewage sludge treatment. Sludge treatment is a research concern, because it combines physical, chemical and biological processes. It is known that sludge must be separated from wastewater to be treated—stabilised, thickened and disinfected—before taking it to its final disposal, mainly as landfill or compost.

Subjects that are also of particular concern for researchers include special treatments such as gasification, biofuel, anaerobic digestion and pyrolysis.

Recycling of waste is essential for the waste hierarchy to function and is the subject of research and improvement, which is why the keyword ‘recycling’ is widely discussed.

The study of ‘landfills’ is also a recurring theme, as is that of ‘environmental impact’, because researchers focus their concern on the non-degradation of nature as well as treatments that are safe for human health.

It is important that new researchers use valorisation or waste-to-energy articles to be at the forefront of this dynamic driving issue, especially in their theses or other studies, to form their theoretical frameworks.

According to the analysis carried out in relation to the journals, *Waste Management* is the most published and cited journal in the field of incineration and valorisation.

## 5. Conclusions

The methodology used is effective because it can analyse, from a longitudinal point of view, how concepts evolve, and by focusing on a specific period, it is able to determine which topics are driving themes and important for the research field. This methodology also informs us about which topics are of marginal importance because they are highly specialised or peripheral. In addition, the methodology can detect emerging themes and declining themes.

In conclusion, it can be said that giving waste a second life is essential. Being able to create a true circular economy in which zero waste is reached is hopefully achievable in the near future. To achieve this, the hierarchy of waste must be respected, which indicates that with regard to waste, work must be done in this order: prevention, minimisation, reuse, recycling, recovery, and elimination.

In terms of recovery, energy recovery (waste-to-energy) must be considered, among which is incineration. The concept of incineration has had great public opposition, and the literature shows that this concept has been transformed towards a more generic concept such as valorisation or waste-to-energy.

### 5.1. Theoretical Implications

This article presents a significant theoretical contribution in the field of research on waste management, organising the knowledge generated in this area to date and detecting the driving themes in which the research will be structured in the coming years. From a theoretical point of view, all the articles from the Web of Science (WoS) related to incineration and valorisation were analysed, which provides clarity due to the thematic evolution maps that help us to better understand the longitudinal progression of the research field.

This study offers an important theoretical contribution to the field of research on the valorisation of waste, by expanding the existing knowledge. The use of co-word analysis allowed us to conduct a study of the valorisation and incineration literature to examine this field, its structures, patterns of influence, and propose future research directions. Furthermore, the study presents SciMAT as a good tool for performing longitudinal bibliometric analyses.

In addition, the longitudinal bibliometric analysis is presented as very suitable to carry out this type of analysis, which has not been performed in this field to date.

### 5.2. Practical Implications

From a practical point of view, the findings of this research are of substantial interest to different stakeholders such as policymakers, consumers and companies, among others. The benefits in this field for most stakeholders represent a considerable benefit to humanity.

This article reflects growing environmental sensitivity and pressure for proper waste management.

The work shows that the research topics at present and in the near future will be focused around some motor topics. They are well developed and important topics to structure the research field. The study shows that the most important motor theme is valorisation. Appreciation is the way. Around this theme, other important themes will revolve such as waste management, incineration, municipal solid waste, environmental impact, among others.

### 5.3. Limitations and Future Lines of Research

This work has some limitations. On the one hand, we have worked with articles included in the WoS Core Collection database. Although it is true that this database is very complete and relevant, without a doubt, other collections could be used in future bibliometric investigations.

Another limitation comes from the management of the software itself, where the researcher must guide the study, through filters in each of the steps, to achieve the necessary precision.

A future line of research could include a co-citation analysis, which would complement this study.

## Figures and Tables

**Table 1:** Number of documents per period.

Number	Period	Number of Documents
1	1987–2010	39
2	2011–2015	55
3	2016–2021	120
	Total	214

**Table 2:** Number of documents ordered by source.

Source	Documents
*Waste Management*	22
*Resources Conservation and Recycling*	14
*Waste and Biomass Valorisation*	11
*Journal of Cleaner Production*	10
*Journal of Hazardous Materials*	9
*Sustainability*	7
*Renewable Energy*	5
*Bioresource Technology*	4
*Construction and Building Materials*	4
*Journal of Material Cycles and Waste Management*	4
*Energies*	4
*International Journal of Life Cycle Assessment*	4

**Table 3:** Most cited documents.

Title	Source	Year	Number of Citations
Food waste as a valuable resource for the production of chemicals, materials and fuels. Current situation and global perspective [1]	*Energy & Environmental Science*	2013	524
Possible applications for municipal solid waste fly ash [9]	*Journal of Hazardous Materials*	2003	273
Life cycle assessment of biofibres replacing glass fibres as reinforcement in plastics [8]	*Resources Conservation and Recycling*	2001	222
The crucial role of waste-to-energy technologies in enhanced landfill mining: A technology review [5]	*Journal of Cleaner Production*	2013	209
Food waste generation and industrial uses: A review [20]	*Waste Management*	2015	200
The valorisation of plastic solid waste (PSW) by primary to quaternary routes: From re-use to energy and chemicals [14]	*Progress in Energy and Combustion Science*	2010	197
Automotive shredder residue (ASR): Reviewing its production from end-of-life vehicles (ELVs) and its recycling, energy or chemicals’ valorisation [21]	*Journal of Hazardous Materials*	2011	160
Physical and chemical characterisation of crude meat and bone meal combustion residue: “Waste or raw material?” [22]	*Journal of Hazardous Materials*	2005	131
Valorisation of bark for chemicals and materials: A review [23]	*Renewable & Sustainable Energy Reviews*	2013	124
Study of bio-oils and solids from flash pyrolysis of sewage sludges [24]	*Fuel*	2009	118
Carbon footprint of food waste management options in the waste hierarchy—a Swedish case study [25]	*Journal of Cleaner Production*	2015	107
Advances on waste valorisation: New horizons for a more sustainable society [26]	*Energy Science and Engineering*	2013	88
Environmental sustainability assessment of food waste valorisation options [27]	*Resources, Conservation and Recycling*	2014	87
Laying waste to mercury: Inexpensive sorbents made from sulfur and recycled cooking oils [28]	*Chemistry—A European Journal*	2017	83
Value-added chemicals from food supply chain wastes: State-of-the-art review and future prospects [29]	*Chemical Engineering Journal*	2019	63
Manufacture of hybrid cements with fly ash and bottom ash from a municipal solid waste incinerator [30]	*Construction and Building Materials*	2016	62
Recycling and recovery of post-consumer plastic solid waste in a European context [31]	*Thermal Science*	2012	57
Disposal options for polluted plants grown on heavy metal contaminated brownfield lands—A review [32]	*Chemosphere*	2017	51

**Table 4:** Cluster properties.

	Cluster	Centrality	Centrality Range	Density	Density Range	Documents Count	h-Index	Average Citations	Sum Citations
1987–2020	Residues	141.60	1.00	53.59	0.80	7	4	45	315
	Resource recovery	123.03	0.80	130.90	1.00	3	3	161	483
	Waste	109.85	0.60	31.11	0.40	8	7	39.25	314
	Fuel	33.33	0.40	43.75	0.60	2	2	70.5	141
	Municipal solid waste	23.03	0.20	13.33	0.20	2	1	2.5	5
2011–2015	Incineration	200.12	1.00	42.60	0.44	25	14	29.96	749
	Anaerobic digestion	156.89	0.89	66.99	0.78	8	7	53.125	425
	Pyrolysis	130.62	0.78	25.09	0.22	7	4	15.857	111
	Waste	100.40	0.67	37.99	0.33	9	8	56.889	512
	Environmental impact	94.29	0.56	52.01	0.56	9	9	50.889	458
	Lactic acid	51.82	0.44	20.83	0.11	3	2	241.667	725
	Silica	33.74	0.33	60.00	0.67	3	2	9.333	28
	Fast pyrolysis	20.01	0.22	81.25	0.89	1	1	124	124
	Construction	15.65	0.11	150.00	1.00	1	0	0	0
2016–2021	Valorisation	197.17	1.00	39.92	0.90	93	18	9.688	901
	Recycling	129.97	0.90	13.20	0.30	12	6	13.167	158
	Ash	123.86	0.80	30.33	0.80	27	8	9.852	266
	Electricity	102.02	0.70	58.44	1.00	5	2	18.6	93
	Optimisation	90.14	0.60	29.55	0.70	7	3	5.714	40
	Pyrolysis	88.60	0.50	12.93	0.20	10	3	6.5	65
	Wastewater treatment	86.48	0.40	13.76	0.50	13	6	9.538	124
	Systems	59.65	0.30	13.56	0.40	4	3	11	44
	Products	29.42	0.20	12.50	0.10	3	2	10.333	31
	Pulp	14.61	0.10	21.88	0.60	2	1	4	8

## Data Availability

Not applicable.
